# Sanitary Entombment

**Published:** 1890-02

**Authors:** 


### BOOK NOTICES.

Sanitary Entombment; The Ideal Disposition of the Dead. By the Rev. Charles R. Treat, Rector of the Church of the Archangel, New York City.

This interesting pamphlet, as its name denotes, is a plea for an improved or "ideal" method of putting away the dead. This ideal method is nothing more or less than the method of nature—dessication or drying.

The proposition is to build, "as the unit of construction," a sepulchre, constructed in such manner that dried air should enter, and after circulating over the corpse be withdrawn, laden with moisture and the exhalations of the body, and conducted to a furnace where these would be disposed of effectually.

These sepulchres would be gathered together in large buildings, similar to the "Campo Santo" at Pisa. They might be of plain architecture, or they might be of magnificent and expensive design.

The pamphlet before us gives cuts of designs that are superb specimens of architecture and strongly suggestive of that lovely miracle of architecture, the Alhambra.

Such a method of burial would do away with the pest-breeding churchyard; would meet the objections of those who revolt at cremation, and, generally speaking, would give the following advantages:

There is no mutilation, no substitution of foreign substances for human flesh as in embalming; no preserving "the semblance of the human form so long that sentiment is shocked and a due return of material humanity to the elements that gave it birth prevented;" no decomposition with its awful products polluting the atmosphere and befouling the streams of water used for drinking purposes. No disease germs to rise and start pestilence in the cities.

On the other hand, it means perpetual care of the dead; it protects from the possibility of being buried alive, since these sepulchres are to be fitted with electrical apparatus that in the event of reviving life would give the alarm; it protects the dead from theft; "protects the living from exposure whilst paying the last duties to the dead; meets the urgent sanitary demand that the dead shall not endanger the living; and meets the medico-legal demand that the evidence of crime shall not be destroyed."

